# Heterogeneity in the Analysis of the ALSFRS-R in ALS Clinical Trials and its Effect on the Validity and Precision of Trial Conclusions

**DOI:** 10.1212/WNL.0000000000214937

**Published:** 2026-04-21

**Authors:** Daphne N. Weemering, Jordi W.J. van Unnik, Angela Genge, Leonard H. van den Berg, Ruben P.A. van Eijk

**Affiliations:** 1 Department of Neurology, UMC Utrecht Brain Centre, University Medical Centre Utrecht, the Netherlands;; 2 ALS Centre of Excellence, Montreal Neurological Institute-Hospital, Canada; and; 3 Biostatistics and Research Support, Julius Centre for Health Sciences and Primary Care, University Medical Centre Utrecht, the Netherlands.

## Abstract

**Background and Objectives:**

Disability rating scales play a pivotal role in clinical trials, but there is a notable lack of guidance on how to analyze these scales. Using amyotrophic lateral sclerosis as a case study, our aim was to explore how disability rating scales have been analyzed in completed clinical trials and to assess how these different approaches influence both the risk of false-positive findings and the statistical power to detect true treatment effects.

**Methods:**

We searched PubMed and Embase to systematically identify randomized, placebo-controlled clinical trials using the revised ALS functional rating scale (ALSFRS-R) as primary end point, with ≥20 randomly assigned patients and ≥12-weeks of follow-up. Data were extracted on the statistical analysis approaches and strategies for handling missing data. Variability in statistical methods was mapped to the various research questions that the trials aimed to address. A simulation study assessed how each statistical method influenced validity (false-positive rate) and precision (statistical power), using the Ceftriaxone trial data set to model a realistic trial scenario.

**Results:**

Our analysis included 45 randomized clinical trials, comprising a total sample size of 7,338 patients, and identified 39 distinct statistical methods using a mixture of longitudinal and cross-sectional techniques. Most trials (55.6%) did not use all available (longitudinal) ALSFRS-R measurements, resulting in suboptimal utilization of patient data and reduced statistical precision. Applying the different statistical methods to the same trial data set resulted in large differences in the estimated treatment effect size, ranging from a negative 1.33 to a positive 2.33 SD difference. Among the methods used, 38.9% (95% CI 24.8%–55.1%) were at risk of increasing false-positive rates, potentially contributing to the erroneous advancement of ineffective treatments. Statistical power of valid strategies varied widely, ranging from 17.9% to 78.2%.

**Discussion:**

Our results demonstrate considerable variability in statistical methods, with the choice of method able to influence the estimated treatment effects, potentially resulting in misleading conclusions and uncertainty about treatment effects. This limits the interpretability and comparability of clinical trials and influences clinical decision-making and drug development. Establishing statistical consensus recommendations could improve the utility of disability scales in clinical trials and accelerate progress toward effective therapies for neurodegenerative diseases.

## Introduction

Disability rating scales play a pivotal role in randomized clinical trials (RCTs), in evaluating the effect of experimental drugs on the daily lives of people with a neurodegenerative disease. These outcome measures track changes in symptoms, functionality, or quality of life over time. As such, they provide complementary information alongside survival outcomes on the clinical relevance and risk-benefit profile of new therapies—a priority also emphasized by regulatory agencies.^1,2^ When used consistently and analyzed carefully, these measures enable informed, patient-centered decision-making regarding treatment selection in clinical practice. This has led to the development of disease-specific rating scales for various neurodegenerative diseases,^3-5^ which have rapidly gained prominence as primary efficacy outcomes in clinical trials, supporting the approval of therapeutic agents in Alzheimer disease, Parkinson disease, and amyotrophic lateral sclerosis (ALS).^6-8^

There is, however, a notable lack of guidance on how to analyze disability scales in clinical trials. A common issue during follow-up is the occurrence of patient deaths or study withdrawals, resulting in incomplete data. Managing missing data pose complex statistical challenges,^9,10^ further complicated by the considerable clinical heterogeneity among patients and the multidimensional nature of neurodegenerative diseases.^11^ Each of these challenges can be addressed in different ways, leading to a wide range of analysis strategies across clinical trials, even when using the same outcome measure.^12^ These inconsistencies complicate the comparisons between studies and the evaluation of new treatments. This variability obscures a clear understanding of the true benefits and risks of novel treatments, potentially leading to false-positive or false-negative findings.^13^

In response to this gap in guidelines and best practices, a Working Group was established with the goal of harmonizing the analysis of disability rating scales in clinical trials,^14^ using the (revised) ALS functional rating scale (ALSFRS-R) as showcase example. A central objective of the initiative was to provide an overview of the current landscape of analytical methods and to connect the choice of analysis to the specific clinical question that a trial seeks to answer, known as the trial's estimand.^15^ In this context, this study's aim was to characterize current analytical practices for the ALSFRS-R in randomized controlled trials and to assess how these choices influence the validity and precision of trial conclusions.

## Methods

This study consisted of 2 parts. In the first part, we conducted a systematic review to identify and categorize the statistical methods used to analyze the ALSFRS-R in clinical trials for ALS, using the estimand framework to map how different approaches align with specific clinical questions. In the second part, we evaluated the effect of these statistical approaches on false-positive and false-negative findings by reanalyzing a completed clinical trial.

### Search Strategy and Selection Criteria

The systematic review was conducted based on the principles of the Cochrane Handbook for Systematic Reviews of Interventions and reported according to the Preferred Reporting Items for Systematic reviews and Meta-Analyses (PRISMA) 2020 guidelines.^16,17^ Only RCTs of patients diagnosed with ALS, according to the (revised) El Escorial, Awaji, or Gold Coast criteria, were considered.^18-20^ Clinical trials were eligible if they randomized at least 20 patients to exclude very small, early phase studies, evaluated a disease-modifying therapy, included a placebo-controlled period of at least 12 weeks, and used the ALSFRS-R as primary end point (either standalone or combined with survival). Both shorter (phase 2) and longer (phase 3) trials were included to describe and contrast the statistical methods historically applied across different trial contexts. The analysis focused on the statistical methods reported in each trial rather than the trial outcomes, and differences in objectives or design did not affect our analysis. Studies focusing on symptomatic treatments, devices, or lifestyle interventions were excluded. Studies using single-arm, crossover, or externally controlled designs, or those starting enrollment before January 1999, were also excluded. Only articles published in English were considered. To identify relevant studies, the PubMed and Embase databases were searched in November 2023 using the terms “amyotrophic lateral sclerosis” or “motor neuron disease” and “clinical trial*”. The full details of the search are provided elsewhere.^21^ This work represents a secondary analysis of a previously published systematic review protocol and was therefore not registered separately in a public database.

### Data Extraction

Data from included studies were extracted by 2 reviewers (DW and JvU) using a standardized data extraction sheet. Data were organized into 4 categories, namely: general study descriptives, information about study design, reasons for dropout and handling missing data, and statistical analysis methods and covariate adjustment (eMethods 1). We did not collect or analyze patient-level or aggregate trial outcomes; the extraction focused on the reported statistical analysis methods for each trial. DW conducted the initial data extraction, with a random sample of 25% being verified by JvU. Discrepancies were resolved through discussion; a third reviewer (RvE) was available when no consensus could be reached. If substantial discrepancies were identified, an additional 25% sample was planned for independent verification. Data extraction was supplemented with information from the trial's protocol, statistical analysis plan (SAP), or trial registry databases (e.g., ClinicalTrials.gov or EudraCT). In case of conflicting information between data sources, the data used were those that aligned best with the results reported in the trial publication.

### Classification of Study Conclusion and Statistical Methods

For each study, we determined whether the study drug was reported to have a beneficial, futile, or harmful effect on the ALSFRS-R based on the study's conclusion statement. Studies with a futile or harmful effect were grouped together as “futile,” whereas beneficial effects were grouped together as “superior.” The statistical analysis method was categorized based on the summarization of the primary outcome measure (e.g., change from baseline, proportion of responders, or mean rank score), analysis approach, and covariate adjustment. The analysis method was further classified according to its underlying assumptions. For example, analyses of continuous, normally distributed data—such as *t* tests and analyses of (co)variance (AN(C)OVA)—were grouped under “linear regression,” whereas analyses of binary outcome data, such as odds ratios and chi-square tests, were grouped under “logistic regression.” Handling of missing data was divided into 2 categories: missing data because of death and missing data because of nondeath dropouts. Analyses using mixed effects models, such as random-effects models and mixed models for repeated measures (MMRMs), do not address missing data through explicit imputation. Instead, they account for missing data implicitly during estimation. We refer to this approach as an implicit missing data strategy (MDS). Additional details on categorization definitions are provided in eMethods 2.

### Estimand Assignment

The clinical question addressed by each study was categorized according to the estimand framework outlined in the International Council for Harmonisation of Technical Requirements for Pharmaceuticals for Human Use (ICH) E9(R1) addendum.^15,22^ Strategies were classified based on how the statistical analysis addressed the terminal—or truncating—intercurrent event of death, assigning each study into 1 of 4 strategies: (1) hypothetical, (2) principal stratum, (3) while alive, or (4) composite. These strategies shape the clinical question being addressed, and consequently the treatment effect being estimated. The hypothetical strategy estimates the treatment effect at a specific time point hypothesizing that all patients survived, for example, by imputing a hypothetical score for any deceased patients and calculating a mean difference. Similarly, the principal stratum strategy focuses on those patients who were alive at a specific time point, regardless of treatment. The mean difference is therefore estimated in the patients who are expected to survive, for example, by excluding outcome data of those who died from the analysis (i.e., complete case analysis). By contrast, the while-alive strategy analyses the outcome data collected before death, thereby using all available measurements from each patient. The treatment effect is often expressed as the mean difference in progression rates over a specific time period or until death, whichever comes first. This approach is particularly suitable for longitudinal data, with random-effects models frequently used to estimate the progression rate over time. Finally, the composite strategy integrates survival with ALSFRS-R scores and estimates the combined effect of treatment on function and survival, typically achieved by ranking patients based on survival time and ALSFRS-R scores.^23^ This strategy might be of particular interest for ALS clinical trials with relatively long study periods in which the treatment effect is relevant both on survival and on ALSFRS-R.^24^

### Statistical Analysis

Study characteristics were summarized using frequencies and percentages for categorical variables, and medians and first and third quartiles for continuous data. Differences between categorical variables were assessed using Fisher exact test, with 95% CIs for single proportions calculated as the Wilson CIs. Differences between continuous variables were evaluated using the Mann–Whitney or Kruskal–Wallis tests, as appropriate.

To evaluate the effect of different statistical methods on study findings, we reanalyzed the Ceftriaxone ALS study, a large phase 3 trial with a well-characterized 52-week follow-up duration. This study randomly assigned 513 patients to placebo or ceftriaxone treatment.^25^ Of these, 245 patients (47.8%) had missing data, 104 patients (20.3%) dropped out of the study, and 93 patients (18.1%) died during follow-up. The dropout rate was similar in both treatment groups (19.7% in placebo group and 20.6% in treatment group). We applied each statistical method to the ceftriaxone trial data to investigate the variability in the estimated treatment effects. To facilitate the comparison, we transformed the observed *p* values to *z*-scores, where a *z-*score of ±1.96 corresponds to a *p* value of 0.050. Some of the statistical methods identified could not be fully replicated because of missing covariates or requiring a lead-in period. All adjustments and assumptions are listed in eMethods 3.

For each statistical method, we determined the validity, expressed as false-positive rate or type 1 error, through permutation testing. Specifically, we created hypothetical trials under a scenario in which no treatment effect was present by randomly reassigning patients to the ceftriaxone or placebo group, such that both groups were drawn from the same underlying population. Therefore, any statistically significant treatment effect identified in this setting (*p* < 0.050) represents a false-positive finding attributable to the statistical method (rather than the data). For each statistical method, the false-positive rate was calculated as the percentage of 10,000 permutations in which a statistically significant treatment effect was found. To avoid overly conservative interpretations, two-sided false-positive rates ≤0.10 (i.e., twice the conventional 0.050 threshold) were considered indicative of acceptable validity. This threshold served as a pragmatic benchmark to distinguish methods with substantially inflated false-positive rates from those performing approximately as expected, and does not imply the use of a 0.10 cutoff for hypothesis testing in clinical trials.

To estimate the effect of each method on precision or statistical power, we used a similar permutation exercise with the addition of a hypothetical 20% slowing in ALSFRS-R progression rate for those assigned to treatment. This effect size, in a sample of 500 with 2:1 randomization, yields approximately 80% power when using a random slopes model. Because dropout and death rates are balanced when conducting a permutation exercise, we additionally evaluated a scenario with differential attrition by increasing the attrition rate in the ceftriaxone group to 40% (compared with the original 20.6%). As sensitivity analysis, we repeated the permutation analyses in a smaller phase 2 trial (43 patients, 24-week follow-up)^26^ to confirm that our findings are consistent in trials of shorter duration and smaller sample size. Permutation simulations were further supported by a model-based simulation using the joint-modeling framework.^27^ Further details on the simulation model are provided in eMethods 4 and eTable 1. All analyses were conducted in R (version 4.4.0; 2025). Analysis files and package information are available on GitHub.^28^

### Standard Protocol Approvals, Registrations, and Patient Consents

This study was a secondary analysis of previously collected, fully deidentified data. The original data were obtained from a source in which participants had already provided consent for research, and no new participant contact or interventions were performed.

### Data Availability

The Ceftriaxone ALS study data that support the findings of this study were obtained from the National Institute of Neurologic Disorders and Stroke (NINDS). Restrictions apply to the availability of these data, which were used under license for this study, and are therefore not publicly available. Data may be made available from NINDS on request.

## Results

### Study Characteristics and Study Information Reporting

**Figure 1:**
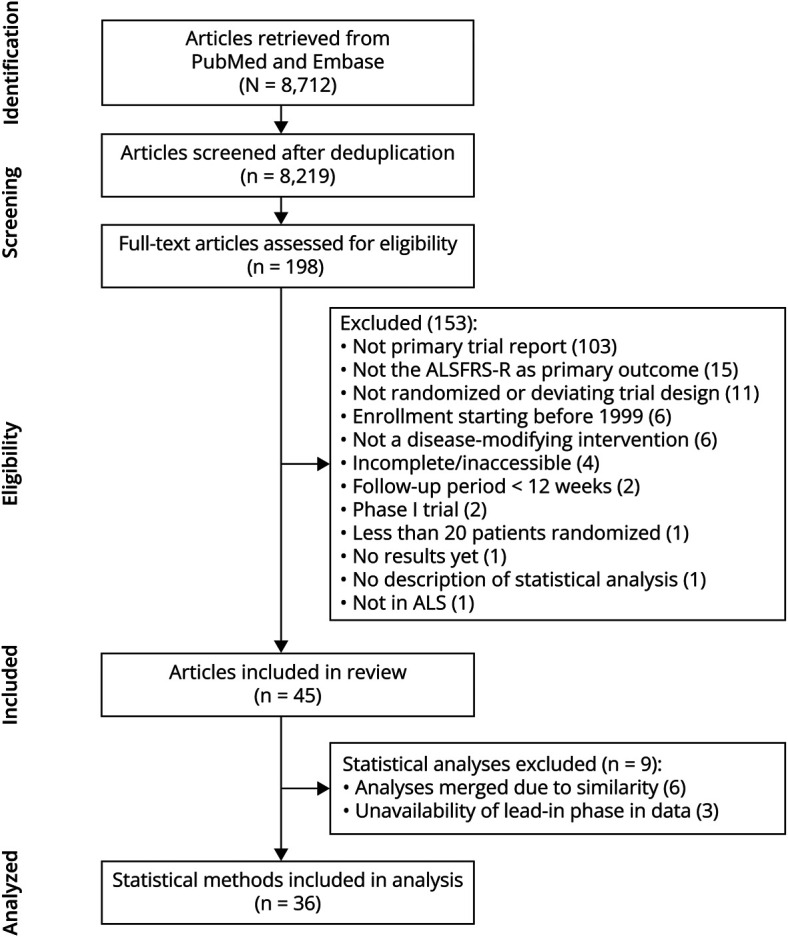
Flowchart of the Selection of Randomized Clinical Trials in ALS Study flowchart of the systematic review and analysis to identify trials with the ALSFRS-R as primary outcome measure. For the analysis part, statistical analysis methods of 6 different trials were merged into 1 method because the analyses were the same; 3 strategies were excluded because of the unavailability of a lead-in phase in the Ceftriaxone ALS and RT001 studies.

The search strategy yielded a total of 8,219 studies, of which 46 were eligible. One study met the eligibility criteria but was excluded as it did not provide any information on the statistical analysis (Figure 1). The remaining 45 RCTs investigated 37 unique therapeutic agents and randomly assigned a total of 7,338 patients. The study characteristics are presented in Table 1; a complete reference list of the included studies is provided in eAppendix 1. Overall, 15 RCTs (33.3%) had a study protocol or SAP publicly available; 35 RCTs (77.8%) concluded a futile (or harmful) treatment effect on the ALSFRS-R. Both superior and futile trials included a similar number of patients, but the futile trials had a longer follow-up compared with superior trials (*p* = 0.046). Of note, only 8 RCTs (17.8%) conducted the analysis in the intention-to-treat (ITT) population, analyzing all randomly assigned patients, whereas a greater number of 28 RCTs (62.2%) used a modified ITT (mITT) population. Across these 28 trials, mITT was defined inconsistently, with 10 different definitions being reported (eMethods 5). Finally, of the 7,338 randomly assigned patients, 7,000 were included in the analysis, excluding a total of 338 patients (4.6%) from the primary analysis.

**Table 1:** Characteristics of Futile and Superior RCTs Using ALSFRS-R as Primary Outcome Measure

	Total (n = 45)	Study conclusion		
		Futile (n = 35)	Superior (n = 10)	*p* Value
Median number of randomly assigned patients	80.0 (42.0–196.0)	80.0 (209.0)	92.0 (100.3)	0.46
Median number of patients analyzed	80.0 (40.0–189.0)	80.0 (210.0)	89.5 (103.5)	0.31
Median follow-up duration in wk	24.0 (24.0–48.0)	28.0 (24.0)	18.0 (8.0)	0.046
Primary analysis population, (%)				0.68
ITT	8 (17.8)	7 (20.0)	1 (10.0)	
mITT	28 (62.2)	22 (62.9)	6 (60.0)	
Other	9 (20.0)	6 (17.1)	3 (30.0)	
Protocol or statistical analysis plan available, yes, (%)	15 (33.3)	11 (31.4)	4 (40.0)	0.71
Median number of study sites	10.0 (2.5–30.0)	12.0 (28.0)	5.0 (23.8)	0.43
Randomization ratio, equal, (%)	33 (73.3)	25 (71.4)	8 (80.0)	0.71
Study phase, (%)				>0.99
Phase 2	27 (60.0)	21 (60.0)	6 (60.0)	
Phase 3	18 (40.0)	14 (40.0)	4 (40.0)	
Year of publication, (%)				0.35
Before 2010	5 (11.1)	5 (14.3)	0 (0)	
2010–2019	24 (53.3)	19 (54.3)	5 (50.0)	
2020 onward	16 (35.6)	11 (31.4)	5 (50.0)	
Sponsor, industry, (%)	23 (51.1)	18 (51.4)	5 (50.0)	>0.99
Geographical location, (%)				0.25
USA	12 (26.7)	11 (31.4)	1 (10.0)	
Japan	9 (20.0)	6 (17.1)	3 (30.0)	
International collaboration, European centered	9 (20.0)	8 (22.9)	1 (10.0)	
International collaboration, North American centered	3 (6.7)	3 (8.6)	0 (0)	
Other	12 (26.7)	7 (20.0)	5 (50.0)	

Abbreviations: ITT = intention-to-treat; mITT = modified intention-to-treat; RCT = randomized controlled trial.

Data are n (%) or median (1st—3rd quartile). Futile RCTs refers to trials which concluded to have either a futile or harmful effect; superior RTCs refer to trials which concluded with a beneficial effect. *p* value gives the *p* value for the comparison between futile and superior trials, based on Fisher exact test for categorical variables, or Mann–Whitney U test for continuous variables. In 2 (futile) RCTs, the number of study sites was missing; these values were excluded from the analysis. Phase 2 studies include all trials described as phase 1/2 or phase 2, whereas phase 3 studies includes all trials described as phase 2/3 or phase 3.

### Reporting of Statistical Analysis and Estimand Strategies for Handling Death

**Figure 2:**
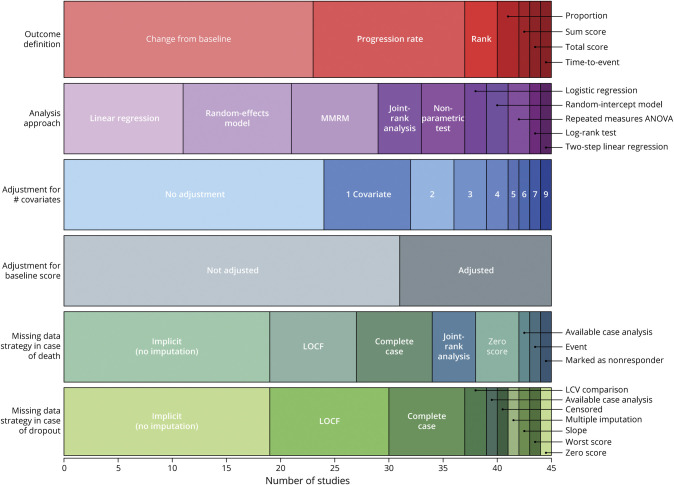
Overview of Components of the Statistical Methods in Included Clinical Trials Variation in elements of the statistical analysis across the included trials. Each stacked bar chart displays the types and frequencies of these elements. ANOVA = analysis of variance; MMRM = mixed model for repeated measures; LCV = last common visit; LOCF = last observation carried forward.

Across the 45 RCTs, we identified 39 unique statistical methods to analyze the ALSFRS-R. These statistical methods were categorized by 6 components: the outcome definition, analysis approach, baseline adjustment, covariate adjustment (other than baseline), MDS in case of death, and MDS for nondeath dropout (Figure 2); a description of each statistical method is provided in eMethods 1. The 2 most frequently used analysis approaches were linear regression (24.4%) and random-effects models (22.2%). More than half of the trials (55.6%) did not make use of all available ALSFRS-R measurements. Implicit or model-based handling of missing data were the most common approach for addressing missing data (42.4%). Half of the trials (51.1%) incorporated 1 or multiple covariate(s) into their analysis, with the baseline score (31.1%) being most frequently used (eFigure 1). In addition, 30 (66.7%) trials stratified the randomization, and of these, only 10 (33.3%) incorporated the stratification factors as covariates into their statistical model. Forty-four RCTs (98.0%) reported at least 1 intercurrent event that resulted in permanent discontinuation of follow-up (eFigure 2). Notably, 36 (80.0%) RCTs applied the same strategy for handling missing values, regardless of whether they were due to death or nondeath dropout.

**Figure 3:**
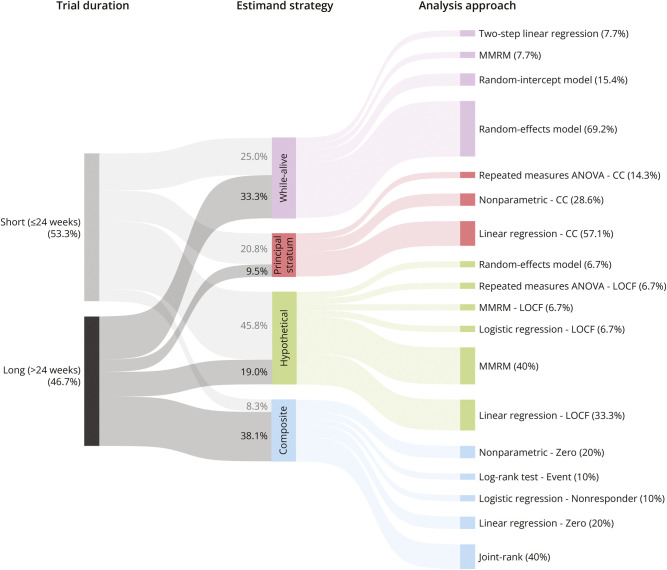
Statistical Methods of ALS Clinical Trials Categorized by Trial Duration and Estimand Strategy Statistical analysis methods of the clinical trials categorized into 4 groups according to the estimand framework, which considers how analyses account for death. The estimand approaches are stratified for short-duration trials (≤24 weeks) and longer trials (>24 weeks), showing a differential preference for the hypothetical and composite strategy. CC = complete case; LOCF = last observation carried forward; MMRM = mixed model for repeated measures.

The different statistical methods were mapped to the different clinical questions that they answer, based on how survival was incorporated in the analysis (Figure 3). Overall, there was extensive variability in the clinical questions that were addressed by each study. In 15 of the 45 trials (33.3%), the hypothetical estimand strategy was used, thereby assuming patient death does not occur in ALS trials. The principal stratum strategy was the least common strategy, applied in 7 trials (15.6%), mostly by restricting the analysis to survivors only. Of note, the estimand strategy varied by study duration (*p* = 0.048, Figure 3), with shorter trials (≤24 weeks) favoring the hypothetical strategy (45.8%), whereas longer trials (>24 weeks) more often accounted for death using composite (38.1%) or while-alive strategies (33.3%).

### Variability in Results of Different Methods

**Figure 4:**
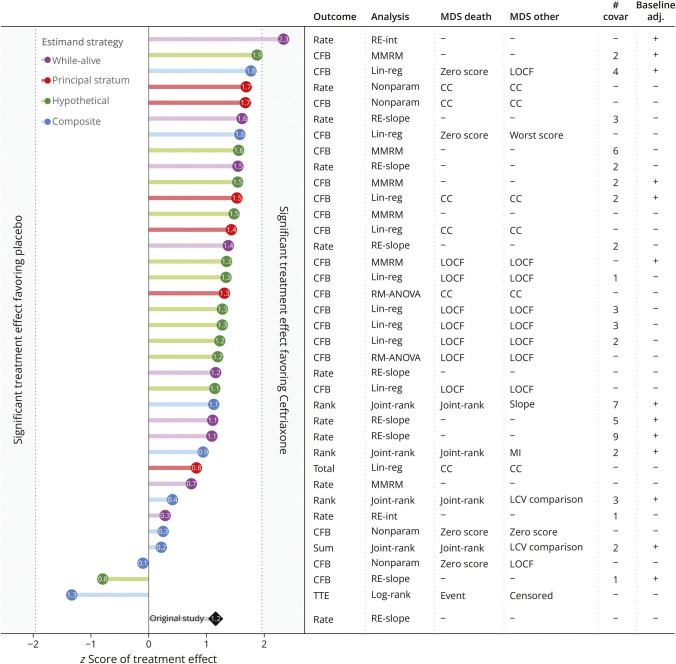
Variation in Treatment Effects Across Statistical Methods in the Ceftriaxone ALS Study Each statistical method was applied to the Ceftriaxone ALS trial, with *p* values transformed to a *z*-score to facilitate between-method comparison. Analysis methods are categorized according to the estimand strategy for missing data (colors), showing a large variability in effect sizes between statistical methods. For missing data strategies, “–” indicates implicit missing data handling (e.g., in random-effects models). adj. = adjustment; CC = complete case analysis; CFB = change from baseline; LCV = last common visit; lin-reg = linear regression; LOCF = last observation carried forward; MDS = missing data strategy; MI = multiple imputation; MMRM = mixed model for repeated measures; nonparam = nonparametric analysis; RE-int = random-intercept model; RE-slope = random-effects model; RM-ANOVA = repeated measures analysis of variance.

**Figure 5:**
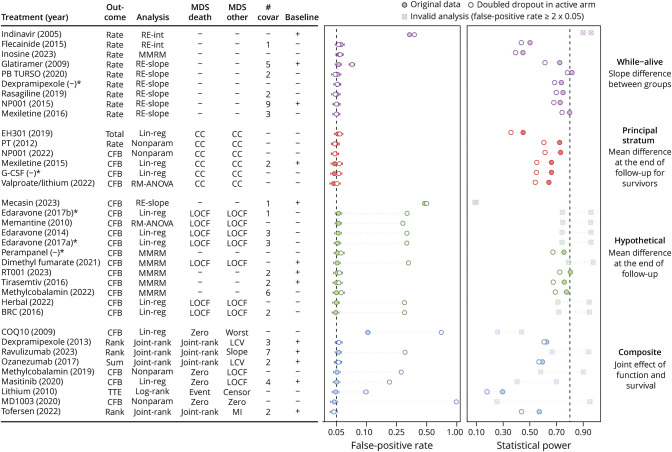
Validity and Precision of Statistical Methods Used in ALS Clinical Trials The left side of the figure displays the validity, expressed as false-positive rate; the right-hand side of the forest plot shows the precision, expressed as statistical power. The filled points indicate the estimates when missing data are balanced and the open points when missing data are unbalanced. Crossed points indicate methods with inflated false-positive rates (≥10%), for which power estimates are not interpretable because the method does not adequately control the false-positive rate. The bars represent the 95% CIs. Estimates are sorted according to the estimand strategy for handling missing data. Analyses using repeated measures ANOVA were adjusted using the Greenhouse–Geisser correction. For missing data strategies, “–” indicates implicit missing data handling (i.e., in random-effects models and MMRMs). * Six trials have been omitted because their analyses correspond to the methods already represented by dexpramipexole, G-CSF, or perampanel. * Edaravone (2017a) is the larger 2017 trial (PMID: 28522181) and edaravone (2017b) is the smaller 2017 trial (PMID: 28872915). BRC = bromocriptine mesylate; CC = complete case analysis; CFB = change from baseline; COQ10 = coenzyme Q10; G-CSF = granulocyte-colony stimulating factor; LCV = last common visit; lin-reg = linear regression; LOCF = last observation carried forward; MI = multiple imputation; MMRM = mixed model for repeated measures; MDS = missing data strategy; nonparam = nonparametric analysis; PB TURSO = sodium phenylbutyrate-taurursodiol; PT = pioglitazone HCI and tretinoin; RE-int = random-intercept model; RE-slope = random-effects model; RM-ANOVA = repeated measures analysis of variance.

Of the 39 different statistical methods, 3 were excluded because of the use of a lead-in period; the remaining 36 methods were applied to the Ceftriaxone ALS Study to assess their statistical performance. Figure 4 depicts the variability in estimated treatment effects with z-scores ranging from −1.33 (harmful) to +2.33 (effective). The validity and precision of the different statistical methods are presented in Figure 5. Four methods (11.1%, 95% CI 4.4%–25.3%) failed to maintain a false-positive rate (type 1 error) when missing data were balanced across treatment groups. Under the scenario with increased dropout in the active group, 19 methods (52.8%, 95% CI 37.0%–68.0%) failed to control false-positive rates below 5%, and 14 methods (38.9%, 95% CI 24.8%–55.1%) exceeded the 10% tolerance boundary; these methods were deemed invalid because of excessive inflation of the type 1 error rate. Inflation of the false-positive rate was in all cases either because of model misspecification or a biased imputation method. Invalid methods were used in 25.0% of studies published before 2017 (median publication year) and in 40.9% of those published afterward, *p* = 0.34. Similarly, studies with a futile conclusion used invalid methods in 27.3% of the cases, whereas studies with a superior conclusion used such methods in 55.6% cases, *p* = 0.13.

For statistically valid analysis methods, defined as those keeping the false-positive rates below 10%, statistical power ranged from 29.5% to 81.6% when dropout was balanced across treatment groups, and from 17.9% to 78.2% when dropout was unbalanced. The highest power was observed in analyses that leveraged all available data points using either a random slopes model (while-alive strategy) or an MMRM (hypothetical strategy), with both approaches yielding a median power estimate of 68.3% (IQR = 14.0% and 2.4%, respectively). Of note, power varied significantly across estimand strategies, with the composite strategy having the lowest precision. This is partially due to our simulations modeling an effect on function only. Sensitivity analyses using a model-based simulation showed that the composite and while-alive statistical methods perform similarly when treatment affects both function and survival (eFigure 3). Furthermore, the high variability among composite methods suggests that modeling choice can greatly influence statistical power of this strategy. The worst-performing composite approach included a time-to-6-point decrease in ALSFRS-R or death analysis using a log-rank test. Sensitivity analyses using the smaller phase 2 trial data showed similar relative differences (eFigure 4).

## Discussion

In this study, we examined the current state of statistical analyses used in RCTs for ALS by reviewing 45 RCTs and identifying 39 distinct methods to analyze the primary disability rating scale, the ALSFRS-R. Of these methods, 38.9% were at risk of inflating false-positive rates well above nominal levels. Of importance, we demonstrate that even randomized trials, that are otherwise well-designed for sample size, duration, and conduct, can yield biased or misleading conclusions when suboptimal statistical analysis strategies are applied. Even among statistically valid methods, there was substantial variability in statistical power. These inconsistencies can mask true treatment effects or falsely suggest benefit, thereby undermining the translation of promising drugs into successful clinical trials or erroneously advancing ineffective therapies, a challenge also observed across neurologic conditions, where inconsistent or suboptimal statistical practices have limited successful translation.^29,30^ Recent negative phase 3 trials exemplify this translational gap.^31^ Similar challenges have been reported in other disease areas, such as oncology, where clinical trials frequently rely on longitudinal patient-reported outcomes or functional scales and where considerable variability and limited guidance in statistical analysis have been described.^12,32^ These issues complicate comparisons across trials, hinder regulatory decision-making, and lead to inefficient use of limited resources, funding, and time. Our findings underscore the need to develop consensus recommendations and to harmonize statistical approaches for analyzing disability rating scales in clinical trials for neurodegenerative diseases.

Our results highlight insufficient adherence to regulatory guidance documents such as the ICH E9 (R1) addendum and to consensus recommendations in the statistical analysis of ALS clinical trials.^22^ These issues have remained consistent over time and may not be unique to ALS, as similar challenges have been reported in other therapeutic areas.^12,33^ Common issues include deviations from the intention-to-treat analysis population, such as selecting patients based on events after randomization or survival time, failure to account for stratification factors used during randomization, omission of baseline values as covariates in the primary analysis, and the use of inefficient analytical methods that ignore available longitudinal data or important prognostic covariates.^29^ Moreover, in only a few trials the study protocol or SAP was publicly available, limiting transparency and reproducibility—principles emphasized in academic and research-oriented guidelines such as CONSORT.^34^

Most notably, despite the clear evidence that inconsistencies in statistical methods can influence trial conclusions and lead to discrepant results,^35,36^ our findings reveal the continued use of statistically invalid approaches in a substantial proportion of studies. This includes the persistent application of last observation carried forward (LOCF) to handle dropout,^37^ and the use of inappropriate model assumptions, such as assuming uniform progression rates in a disease that is inherently progressive and heterogeneous.^38^ Notably, LOCF was used in the pivotal Edaravone study,^39^ which has received regulatory approval in some countries, and as shown by our results, this LOCF approach is susceptible to overstating treatment effects. More broadly, as illustrated by our results, the variability in statistical methods may not solely be due to a lack of adequate guideline adherence and poor practice, but also driven by differences in the clinical questions investigators have sought to answer. For example, when estimating treatment effects on functional decline while patients are alive, longitudinal mixed-effects models that leverage all available ALSFRS-R measurements provide greater precision and better control of false-positive rates than single-point or imputation-based analyses. Underreporting and inconsistent terminology may have further exacerbated this variability, as it was often unclear which specific analytical methods were used in a given clinical trial.

Clearly, given the prominent role of disability rating scales in confirmatory trials across many neurodegenerative diseases, and the variability in statistical methods observed across studies, a consensus approach is warranted. Although our analyses did not identify significant trends over time, the continued use of invalid or imprecise methods in some studies underscores the importance of clear recommendations for the statistical analysis of disability scales. A natural starting point would be to recommend statistical methods tailored to the various clinical questions or estimands of interest. International consensus on how each estimand should be addressed, along with recommendations for reporting to enhance transparency, would make an important contribution to improving the rigor and reproducibility of trials that use disability scales as primary end points, and may facilitate greater adherence to regulatory recommendations in practice.^15,22^ To develop such “estimand-stratified” recommendations, comparative evidence from simulation studies will be essential. These studies can evaluate the operating characteristics of different statistical approaches, such as power, bias, and false-positive rates, and help clarify tradeoffs between methods, including when 1 approach may be preferred more than another.

Several limitations of our study should be acknowledged. Our results provide initial insights into the relative efficiency of different statistical methods for each estimand, but they are not exhaustive in defining the tradeoffs between approaches. Rather, our objective was to identify the relative strengths and weaknesses of commonly used approaches, highlighting methods that perform poorly and those that warrant further investigation, rather than to propose specific methodologic recommendations. We evaluated only a limited set of scenarios; assessment of a broader range of settings is essential before guidance for specific trial contexts can be formulated. In particular, special attention should be given to the treatment effect itself—such as interventions that affect not only the disability scale but also the missing data process, including death—as this directly affects the interpretation of certain estimands, such as those based on a composite strategy.^23,24^ In addition, study design plays a pivotal role in shaping the relevance of different missing data mechanisms, for example, attrition because of death or disease progression may be more prominent in longer trials.^40^ Finally, we restricted our analysis to the methods observed in the selected clinical trials. Other statistical approaches, such as joint models that simultaneously account for functional decline and informative missingness, may offer valuable alternatives and warrant further exploration.^27,41^

In conclusion, our study demonstrates that statistical analyses of the ALSFRS-R in clinical trials are highly variable, with many commonly used methods at risk of inflating false-positive rates or reducing statistical precision. These inconsistencies can alter trial conclusions, obscuring true treatment effects or exaggerating apparent benefits, thereby complicating clinical interpretation and decision making. This has direct implications for researchers and policymakers: suboptimal analyses can distort the estimated treatment effect, potentially leading to the prioritization of ineffective interventions or incorrect assessment of a therapy's benefit-risk profile. In a field marked by a high proportion of negative trials and reliance on disability rating scales for drug development,^42,43^ this variability undermines comparability across studies and confidence in the evidence base, highlighting the need for methodologic consensus. This urgency has been reinforced by recent regulatory evaluations,^44^ which have demonstrated how the choice of statistical strategy and model assumptions can significantly influence trial outcomes and their clinical interpretation. Considering the considerable time, resources, and commitment of trial participants, it is essential to maximize the value of collected data through rigorous statistical methods. To promote broader adoption in industry and ensure alignment with regulatory expectations, formal engagement—through qualification procedures or scientific advice—will be a critical next step. In ALS, this process is already underway through a dedicated working group aiming to improve the analysis and reporting of disability scales,^14^ offering a foundation for broader application in other neurodegenerative conditions.

## Glossary

ALS: amyotrophic lateral sclerosis
ALSFRS-R: revised ALS functional rating scale
ICH: International Council for Harmonisation of Technical Requirements for Pharmaceuticals for Human Use
LOCF: last observation carried forward
MDS: missing data strategy
mITT: modified ITT
MMRM: mixed models for repeated measures
NINDS: National Institute of Neurologic Disorders and Stroke
PRISMA: preferred reporting items for systematic reviews and meta-analyses
RCT: randomized clinical trial
SAP: statistical analysis plan
